# Waste the waist: a pilot randomised controlled trial of a primary care based intervention to support lifestyle change in people with high cardiovascular risk

**DOI:** 10.1186/s12966-014-0159-z

**Published:** 2015-01-16

**Authors:** Colin Greaves, Fiona Gillison, Afroditi Stathi, Paul Bennett, Prasuna Reddy, James Dunbar, Rachel Perry, Daniel Messom, Roger Chandler, Margaret Francis, Mark Davis, Colin Green, Philip Evans, Gordon Taylor

**Affiliations:** University of Exeter Medical School, St Luke’s Campus, Magdalen Road, Exeter, EX1 2LU UK; University of Bath, Claverton Down, Bath, BA2 7AY UK; School of Medicine and Public Health, University of Newcastle, University Drive, Callaghan, NSW 2308 Australia; Greater Green Triangle University Department of Rural Health, Flinders and Deakin Universities, PO Box 423, Warrnambool, VIC 3280 Australia; Bath, Gloucester, Swindon, Wiltshire Area Public Health Team, Public Health England, 1st Floor Bewley House, Marshfield Road, Chippenham, Wiltshire SN15 1JW UK; Waste the Waist Service User Advisory Group, c/o Colin Greaves, University of Exeter Medical School, St Luke’s Campus, Magdalen Road, Exeter, EX1 2PU UK; Centre for Exercise, Nutrition and Health Sciences, University of Bristol, 8 Priory Road, Bristol, BS8 1TZ UK

**Keywords:** Weight loss, Behaviour change, Diet, Physical activity, Randomised controlled trial, Pilot trial

## Abstract

**Background:**

In the UK, thousands of people with high cardiovascular risk are being identified by a national risk-assessment programme (NHS Health Checks). Waste the Waist is an evidence-informed, theory-driven (modified Health Action Process Approach), group-based intervention designed to promote healthy eating and physical activity for people with high cardiovascular risk. This pilot randomised controlled trial aimed to assess the feasibility of delivering the Waste the Waist intervention in UK primary care and of conducting a full-scale randomised controlled trial. We also conducted exploratory analyses of changes in weight.

**Methods:**

Patients aged 40–74 with a Body Mass Index of 28 or more and high cardiovascular risk were identified from risk-assessment data or from practice database searches. Participants were randomised, using an online computerised randomisation algorithm, to receive usual care and standardised information on cardiovascular risk and lifestyle (Controls) or nine sessions of the Waste the Waist programme (Intervention). Group allocation was concealed until the point of randomisation. Thereafter, the statistician, but not participants or data collectors were blinded to group allocation. Weight, physical activity (accelerometry) and cardiovascular risk markers (blood tests) were measured at 0, 4 and 12 months.

**Results:**

108 participants (22% of those approached) were recruited (55 intervention, 53 controls) from 6 practices and 89% provided data at both 4 and 12 months. Participants had a mean age of 65 and 70% were male. Intervention participants attended 72% of group sessions. Based on last observations carried forward, the intervention group did not lose significantly more weight than controls at 12 months, although the difference was significant when co-interventions and co-morbidities that could affect weight were taken into account (Mean Diff 2.6Kg. 95%CI: −4.8 to −0.3, p = 0.025). No significant differences were found in physical activity.

**Conclusions:**

The Waste the Waist intervention is deliverable in UK primary care, has acceptable recruitment and retention rates and produces promising preliminary weight loss results. Subject to refinement of the physical activity component, it is now ready for evaluation in a full-scale trial.

**Trial registration:**

Current Controlled Trials ISRCTN10707899.

**Electronic supplementary material:**

The online version of this article (doi:10.1186/s12966-014-0159-z) contains supplementary material, which is available to authorized users.

## Background

The prevention or delay of cardiovascular disease has great potential for both patient benefit and for reducing costs to health services and the wider economy in most developed countries. Cardiovascular disease is a major cause of reduced patient quality of life, chronic functional limitations and mortality [1,2].

Obesity and physical inactivity are strongly associated with the development of cardiovascular disease [3,4]. A wealth of evidence from randomised controlled trials shows that relatively modest changes in weight (2-3Kg) or physical activity (30–60 mins /week of moderate intensity) modify key cardiovascular risk factors (e.g. cholesterol, blood pressure, HbA1c) to a clinically meaningful extent [5-9]. Type 2 diabetes (a major cause of cardiovascular illness) is also preventable through moderate changes in weight and physical activity in people with Impaired Glucose Regulation (IGR) [10-12]. As well as increasing risk for cardiovascular disease and type 2 diabetes, obesity is associated with an increased likelihood of developing kidney disease, fatty liver disease, osteoarthritis, several cancers, hypertension, dementia, depression and sleep apnoea [13]. In the UK alone, if current trends continue, the combined cost to the National Health Service and to UK society of obesity related illness is forecast to reach £49.9 billion ($82 billion) per year by 2050 [1].

In England, the Department of Health recently began a national programme (NHS Health Checks) to screen all adults aged 40 to 74 to identify and treat high cardiovascular risk [14]. NHS Health Checks should include brief lifestyle advice and more intensive interventions for people with IGR and people with a 10-year cardiovascular risk of 20% or more (calculated using a risk-scoring algorithm and clinical data on risk factors [15]).

However, few of the services that have been used and evaluated to date in the UK, such as Exercise on Referral, Slimming on Referral and group walking schemes have good evidence of either effectiveness or cost-effectiveness [16-19]. This may be because, in practice, these interventions often lack a sound theoretical basis, do not apply up to date evidence on supporting behaviour change [13,20-24] and lack adequate intensity [25,26]. Hence, more sophisticated interventions, which draw on the developing international evidence base for supporting behaviour change are urgently needed.

In prior work [27], we used Intervention Mapping techniques [28] to develop the Waste the Waist intervention. This included extensive literature searching to identify evidence-based practice [25], and assessment of the needs of multiple stakeholders (service users, potential service providers and NHS collaborators). The intervention was designed to have the potential for delivery on a large scale, a cost which is acceptable to primary care stakeholders and to include intervention components recommended in evidence-based guidelines for supporting behaviour change [13,20,26,29].

This paper presents data from a pilot study designed to assess the acceptability and feasibility of the Waste the Waist intervention and of the methods and procedures needed to conduct a full-scale randomised controlled trial and to explore the potential effectiveness of the intervention for supporting weight loss. Further data testing the process model underpinning the intervention and qualitative feedback on acceptability, feasibility and intervention fidelity are reported elsewhere.

## Methods

### Design

A randomised controlled pilot study with nested quantitative and qualitative process evaluation.

### Participants

We recruited people aged 40–74 with a body mass index (BMI) of 28Kg/m^2^ or more *and* with high cardiovascular risk. High cardiovascular risk was defined as any combination of a) a ten-year cardiovascular risk score of 20% or more using either the Framingham [30] or QRISK2 algorithm [15] (these algorithms calculate the risk of future cardiovascular events from clinical data on BMI, blood pressure, cholesterol and other cardiovascular risk factors) b) Impaired Glucose Regulation, defined as either a 2-hour glucose of 7.8 to 11.0 mmol/l (Impaired Glucose Tolerance) or a fasting plasma glucose of 6.1 to 6.9 mmol/l (Impaired Fasting Glycaemia) c) having hypertension, hypercholesterolemia, family history of diabetes or heart disease, history of gestational diabetes, or polycystic ovary syndrome.

We excluded people with existing heart disease, type 2 diabetes or BMI > 45; people who were pregnant or currently using weight loss drugs; people not fluent in English; people with terminal illness and anyone who, in their General Practitioner’s opinion had other co-morbidities which would prevent engagement with the intervention.

All seven of the local practices that were implementing NHS Health Checks in Bath and North and East Somerset at the start of the study were invited to participate and six agreed. These practices provide a range of socio-economic status and ethnic mix which is representative of the Bath and North and East Somerset area. However, this area has limited ethnic diversity, compared with the UK nationally. Potentially eligible participants were identified either from their NHS Health Check test results or by searching practice databases for field codes representing the inclusion /exclusion criteria. Participants’ GPs checked their records for exclusion criteria.

#### Protocol deviation

It is worth noting that our original protocol required a minimum BMI of 30 (and 27.5 for S Asians), but we reduced this to 28 to simplify database searching (practice databases do not record ethnicity) and to facilitate recruitment of sufficient numbers to make up an intervention group in each participating surgery.

### Sample size

The sample size was calculated to provide realistic estimates (and confidence intervals (CIs)) for the recruitment and study completion rates. Based on an expected 30% recruitment rate, in order to estimate the recruitment rate with 95% confidence intervals of +/−5%, we needed to approach 323 people (and recruit around 100). A recruited sample of 100 participants would enable estimation of study completion rates with 95% CIs between +/−9.1 and +/−5.9% (for completion rates of 70 to 90%). To provide reasonable confidence that recruitment would be achievable in a range of practices, we recruited from six general practices.

The primary aim of this study was not to detect differences between groups. However, with 100 participants, we would have 80% power to detect a difference of 3Kg or more in weight loss between groups, assuming a standard deviation of 5.4Kg (which is typical in weight loss trials [31,32]).

### Measures

For the pilot study, the main measures of interest were the recruitment rate and study completion rate (the proportion providing data at 12 months). Intervention attendance (mean number of sessions attended and the proportion attending five or more of the nine sessions) was also measured. All outcome measures to be used in the main trial were also taken, as follows:

#### *Primary outcome* (for the main trial)

Change in weight in Kg at 12 months, measured on calibrated scales.

#### Secondary outcomes

We measured physical activity (mins /day of at least moderate intensity, mins /day of sedentary time, steps per day and total accelerometer counts) using Actigraph GT3XE accelerometers. We measured resting systolic and diastolic blood pressure (taking the lowest of three measurements in the right arm, following at least 3 minutes seated); fasting plasma glucose; fasting LDL, HDL and total cholesterol; triglycerides; glycosylated haemoglobin (HbA1c); liver function tests (our clinical liaison specified alanine transaminase (ALT), which is used as a marker of inflammation [33] as the main variable of interest); BMI; waist circumference (mean of three consecutive measurements); dietary intake (using the DINE questionnaire) [34]; and the EQ-5D health-related quality of life measure [35]. Based on clinical risk markers we calculated QRISK2 ten-year cardiovascular risk score [15] and the presence of metabolic syndrome (a composite cardiovascular risk classification based on having a combination of high waist circumference, fasting plasma glucose, blood pressure and /or lipid abnormalities. We used the WHO definition of metabolic syndrome to assess this [36]).

All outcomes were measured on entry into the study and 12 months later. Four months after baseline we assessed weight, physical activity and questionnaire-based measures to identify the immediate (short-term) impact of the intervention on weight and lifestyle behaviours.

#### Demographic data

Using a questionnaire and practice records, we recorded age, gender, level of education (primary school, some secondary school, secondary school up to 16 years, secondary school up to 18 years, additional training, undergraduate university, postgraduate university), smoking status (ever having smoked and current smoking status), area deprivation (Index of Multiple Deprivation derived from postcode and national census data [37]), family history of heart disease or diabetes (in a parent or sibling prior to age 60), and ethnicity.

#### Process evaluation

Both qualitative and quantitative process evaluations were conducted to determine the relative usefulness of different intervention components and identify ways to refine/improve the intervention [38]. These will be reported in detail elsewhere, but briefly incorporated a) a battery of questionnaires at baseline, four and 12 months to assess changes in all the variables targeted by the process model b) semi-structured interviews with a purposive sample of 18 participants and focus groups with our seven intervention provider staff and c) audio-recording of all intervention sessions to allow checking of intervention fidelity.

#### Co-intervention and co-morbidity

Changes in medications or new diagnoses which might have affected weight (such as thyroid problems, or a prescription of metformin) and participation in other lifestyle-related programmes during the study were recorded by self-report and by examination of participants’ medical records by the researcher at the end of the study.

#### Intervention cost

Intervention delivery costs were estimated from timesheets kept throughout the project by the lifestyle coaches combined with data recorded by the researcher (RP) on administration needs and other resource use (e.g. venue costs, pedometers). The primary item of resource use was the lifestyle coaches (contact time and participant-related non-contact time). Other items of resource use were supervisory time, venue costs for intervention delivery, consumables (e.g. pedometers) and initial set-up /training costs for the intervention. Costs for delivery of the intervention are based on unit costs from published sources, and unit costs collected within the study and are expressed as a mean cost per participant.

#### Further information

Practices were asked to provide anonymised data on patient characteristics for all participants who were eligible and who were invited to take part in the study. The data provided were age, gender, blood pressure, smoking status, cholesterol levels, blood glucose levels, body mass index. This allowed us to assess whether the sample recruited was representative of the wider population.

### Procedures and data collection

Potentially eligible people were invited using a joint letter from their family doctor and a researcher to attend a 35–40 minute study recruitment meeting at their GP surgery. Non-responders and non-attenders were sent a reminder letter. At the recruitment meeting, a researcher (RP) explained the study, answered questions, took consent and measured waist circumference, baseline weight and height. Waist-worn accelerometers and baseline questionnaires were distributed, along with instructions on their use. A practice nurse (or phlebotomist) took blood samples for assessment of baseline clinical measures. Where recent (within two months) fasting blood test data were already available, these tests were not repeated. Any people found to have diabetes at this stage were excluded from further participation and appropriate clinical care was initiated. Eligible participants returned two weeks later to return baseline questionnaires and accelerometers, and were then randomised to either the intervention or control group and further study instructions provided.

Four months later, data were collected by the researcher either at the participant’s home or the University, with accelerometers and questionnaires posted out 14 days beforehand. Data collection at 12 months repeated the baseline data collection procedures although questionnaire and accelerometer data collection was arranged by post. Non-responders were re-contacted and offered a home visit from a research nurse, or phlebotomist.

#### Randomisation

After baseline measurements, participants were randomised to the intervention or control group. The sample was stratified by practice and we then used hierarchical minimisation based on (in order of importance) BMI (up to 35 and 35-plus); pre-diabetes status; and gender. This method uses a computerised algorithm to allocate participants in such a way that a stochastic (random chance) element is preserved, but which also achieves balance between groups in relation to key participant characteristics [39]. An internet-based central allocation service, with 24-hour telephone and/or internet access was developed and implemented by the Peninsula Clinical Trials Unit to ensure independent randomisation/minimisation of any possible selection biases. Concealment of group allocation from both researchers and participants was maintained until the point of allocation.

#### Blinding

The researcher and participants were aware of group allocation at the 4 month and 12 month follow up points, but not at baseline. The rest of the research team, including the nurse or phlebotomist taking blood, the participants’ care team and the statistician were blind to group allocation until the allocation codes were unlocked following analysis.

#### Analysis

Recruitment, intervention attendance, measures-completion rates (for each outcome), resource use and costs and the variance in continuous outcomes at 4 and 12 months were reported using descriptive statistics. Accelerometer data were processed using Actigraph Version 6 software and a protocol successfully used in previous studies [40]. As data for moderate-to-vigorous physical activity were highly skewed, these data were log-transformed. Baseline characteristics were compared between groups using independent t-tests for continuous data and Chi-squared tests for categorical variables. To explore the variance further, exploratory analyses of differences between groups were conducted for weight loss and all the secondary outcomes using ANCOVA analyses. Baseline values for the outcome were entered as covariates. Analyses were based on the intention-to-treat principle, with any missing data imputed used the last observation carried forward (LOCF) method. To examine the possible sensitivity of the data to assumptions about missing data, a complete-case analysis was also conducted. The effects of a) the presence of co-interventions and co-morbidities and b) of any patient characteristics that differed significantly between groups at baseline and c) the minimisation variables on the findings were examined in further analyses which entered these variables as covariates.

The study protocol is published on the International Current Controlled Trials Register (ISRCTN10707899) and the study procedures were reviewed and approved by the NHS National Research Ethics Service SW Research Ethics Committee. The results are reported according to CONSORT guidance for reporting of non-pharmacological interventions [41] as outlined in Additional file 1.

### Intervention

**Figure 1:**
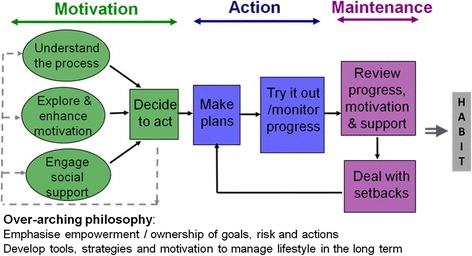
**The Process Model of Lifestyle Behaviour Change [** 44-46]*.*

The development of the “Waste the Waist” intervention is described elsewhere [27] and the intervention content is described in detail in Additional file 2. Briefly, the intervention aimed to encourage weight loss by increasing physical activity, reducing intake of total and saturated fat, increasing fibre intake and other dietary changes (such as reducing portion sizes). Targets were set by participants, but we presented the health benefits of 5% weight loss and of 150 mins per week of moderate activity and suggested that these should be minimum long-term targets for health gain. The intervention was based on the Australian “Greater Green Triangle” (GGT) Programme [42], but we extended the intervention and its theoretical model (the Health Action Process Approach [43]) to include a greater emphasis on social support, self-monitoring and relapse management and the use of coping plans [27]. The intervention processes (Figure 1) involved a) increasing motivation (perceived importance of healthy lifestyle, self-efficacy for achieving healthy lifestyle, perceived risk and outcome expectations); b) making a specific action plan (including plans for social support and for overcoming barriers (coping plans)) and c) supporting maintenance through repeated ‘self-regulatory cycles’ of feedback/reflection, use of self-monitoring and relapse prevention techniques and revision of action plans. There was also a strong emphasis on empowering participants to develop autonomous motivation and to practice skills for lifestyle behaviour change.

To promote sustainability of weight loss we advised participants to make a series of small, achievable changes, rather than dramatic, unsustainable changes. We encouraged participants to prioritise ideas for change that would not detract from their enjoyment of food (for dietary changes) or that would be enjoyable or easy to build into a routine (for physical activity) [47].

#### Training and delivery

The style of delivery was considered to be important and we trained our lifestyle coaches to use person-centred counselling techniques derived from motivational interviewing (open questioning, affirmation, reflective listening, summaries, use of the elicit-provide-elicit (e-p-e) technique for information exchange) [48,49] to promote autonomous motivation and to deliver all of the intervention content. We recruited seven lifestyle coaches from the local community with varied backgrounds and experience, including group-based counselling (n=1), academic qualifications in nutrition or physical activity (n=2) and fitness industry /lifestyle coaching (n=4). A 2.5 day training course was developed and delivered by the co-authors (primarily CG, FG, AS). Supervision meetings were held approximately every two months, where barriers and solutions to delivery were discussed. The lifestyle coaches were given formative feedback from the intervention trainers based on listening to audio-recordings of 1–2 sessions for each pair of trainers.

The Waste the Waist intervention was delivered in local community venues (e.g. community halls, meeting rooms in GP practices after hours). The intervention consisted of four 120-minute group based sessions in the first month to support initial behaviour change, then five 90-minute maintenance support sessions at 1.5, 2, 4, 6 and 9 months after the first session. The total contact time was therefore 13.5 hours spread over 9 months. Groups consisted of 8–12 participants, facilitated by two lifestyle coaches. Participants also received usual GP care.

### Control group

Participants in the control group were posted a standard pack of written information on cardiovascular risk and the effects of diet and physical activity on such risk, in addition to their usual GP care. In the Bath and North and East Somerset area where the study took place, usual care for people with high cardiovascular risk varied considerably, but a number of exercise-on-referral and slimming-on-referral schemes were available and self-referral to commercial weight loss programmes was also possible. Support and encouragement for weight by GPs and practice nurses was also possible, but this was unlikely to consist of more than simple, brief advice. After the collection of 12 month data the control group were offered a condensed (two session) version of the intervention.

## Results

### Recruitment and retention

**Figure 2:**
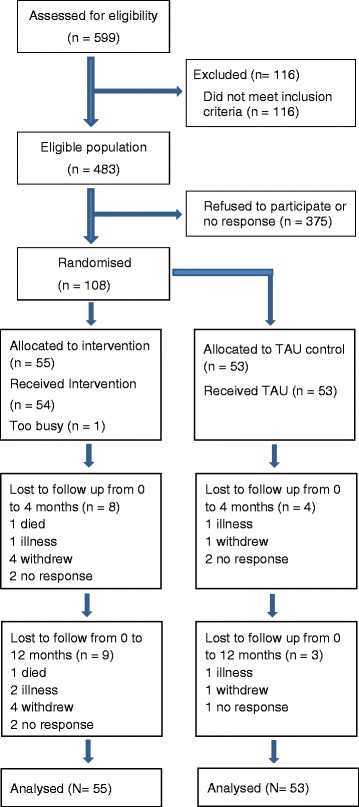
**Flow of participants through the study.**

The flow of participants through the study is shown in Figure 2. Of 483 people approached, 108 (22.4%, 95%CI: 18.6 to 26.2%) were randomised between Feb 2011 and August 2011. Of those randomised, 96 (88.9%, 95%CI: 83.0 to 94.8%) provided weight data at four months and 96 (88.9%, 95%CI: 83.0 to 94.8%) provided weight data at 12 months. The recruitment rate was 15.4 /month and was achieved with one researcher working 3 days per week.

### Sample characteristics

The sample characteristics at baseline are shown in Table 1. The sample was 69% male and 98% white British with a mean age of 65 years. The mean BMI was 33 Kg/m^2^ and 8.5% of the sample had Impaired Fasting Glucose. At baseline, 58% of participants were classified as having metabolic syndrome. The mean risk of a cardiovascular event in the next 10 years (QRISK2 score) was 23%. There were no significant differences between the intervention and control group regarding BMI, pre-diabetes status, gender, or most other baseline variables. However, the intervention group were significantly older than controls, by a mean 2.9 years, had significantly lower diastolic blood pressure by 4.5 mmHg and spent significantly more time being sedentary (42 minutes /day).

**Table 1:** **Sample characteristics at baseline**

**Variable**	**Whole sample** ^**a**^	**N**	**Intervention**	**N**	**Control**	**N**	**p** ^**b**^
Weight (kg)	97.1 (13.4)	108	96.6 (14.0)	55	97.6 (12.8)	53	0.715
BMI (Kg/m^2^)	32.7 (3.1)	108	33.0 (3.2)	55	32.3 (3.0)	53	0.253
Waist (cm)	110 (9.8)	108	110 (10.7)	55	110 (8.8)	53	0.818
Impaired fasting glucose (IFG)	9 (8.5%)	106	3 (5.7%)	53	6 (11.3%)	53	0.488
Male gender	75 (69.4%)	108	36 (65.5%)	55	39 (73.6%)	53	0.407
Age (yrs)	65.1 (7.0)*	108	66.6 (6.4)	55	63.7 (7.4)	53	0.032*
Area deprivation (IMD score)	11.9 (9.5)	108	12.0 (9.2)	55	11.7 (9.8)	53	0.860
*Ethnicity*							
White British	106 (98.1%)	108	54 (98.2%)	55	52 (98.1%)	53	
White Irish	1 (0.9%)	108	0 (0%)	55	1 (1.9%)	53	
White Other	1 (0.9%)	108	1 (1.8%)	55	0 (0.0%)	53	0.368
Systolic BP (mmHg)	138.6 (16.0)	108	137.7 (15.7)	55	139.5 (16.4)	53	0.577
Diastolic BP (mmHg)	82.0 (11.9)	108	79.8 (13.7)	55	84.3 (9.3)	53	0.046*
Fasting glucose (mmol/l)	5.28 (0.56)	106	5.21 (0.50)	53	5.36 (0.60)	53	0.164
Fasting LDL (mmol/l)	3.19 (0.96)	106	3.19 (0.95)	55	3.20 (0.99)	51	0.930
Fasting HDL (mmol/l)	1.37 (0.37)	107	1.36 (0.34)	55	1.39 (0.39)	52	0.672
Fasting total (mmol/l)	5.36 (1.16)	107	5.29 (1.05)	55	5.44 (1.27)	52	0.519
Triglycerides (mmol/l)	1.72 (0.81)	107	1.70 (0.70)	55	1.74 (0.92)	52	0.810
Hba1c (mmol/l)	38.6 (4.29)	106	38.1 (3.5)	54	39.1 (5.0)	52	0.231
Liver function: ALT (IU/l)	28.9 (11.3)	103	27.6 (11.2)	53	30.3 (11.3)	50	0.235
Metabolic syndrome	61 (57.5%)	106	29 (53.7%)	54	32 (61.5%)	52	0.415
QRISK2 Score: 10 year risk (%)	23.1 (10.1)	106	23.6 (9.8)	55	22.5 (10.4)	51	0.556
*Dietary intake (DINE scores)*							
Fat score	31.1 (10.1)	106	30.0 (9.1)	54	32.2 (10.9)	51	0.252
Unsaturated fat score	9.04 (1.80)	105	9.31 (1.65)	54	8.75 (1.93)	51	0.106
Fibre score	36.8 (10.8)	105	36.7 (11.6)	54	37.0 (10.0)	51	0.903
Fruit and Veg score	21.3 (6.9)	105	21.6 (7.3)	54	21.1 (6.6)	51	0.748
*Physical activity*							
MVPA (min /day)	22.0 (19.5)	106	22.5 (22.3)	53	21.6 (16.5)	53	0.813
Sedentary time (min /day)	567.6 (84.1))	106	588.8 (74.4)	53	546.4 (88.5)	53	0.009*
Steps /day	6486 (2757)	106	6420 (3016)	53	6551 (2499)	53	0.807
Counts /minute of valid wear-time (Axis 1)	255.6 (113.1)	106	240.6 (118.4)	53	270.5 (106.6)	53	0.175
EQ-5D VAS overall health score	76.7 (15.9)	107	77.0 (14.9)	55	76.4 (17.0)	52	0.843
*Education levels*							
Up to age 16 or less	50 (46.3%)	108	20 (36.4%)	55	30 (56.6%)	53	
Up to age 18	8 (7.4%)	108	6 (10.9%)	55	2 (3.8%)	53	
Some additional	23 (21.3%)	108	14 (25.5%)	55	9 (17.0%)	53	
Undergraduate or higher degree	27 (25.0%)	108	15 (27.3%)	55	12 (22.7%)	53	0.193

^a^Figures are mean (SD) for continuous data, or N(%) for categorical data. ^b^Analyses are independent group t-tests for continuous data or Chi^2^ for categorical data. *p<0.05.

There were no significant differences between the recruited sample and the wider sample of eligible participants in age, gender or cardiovascular risk score (Table 2). However, the recruited sample had a significantly lower BMI (Mean Diff 1.3 Kg/m^2^; 95%CI 0.6 to 2.0 Kg/m^2^).

**Table 2:** **Comparison of participants and non-participants**

	**Participants** ^**a**^	**N**	**Eligible non-participants**	**N** ^**b**^	**p-value** ^**c**^
**Age**	65.1 (7.0)	108	65.0 (7.3)	276	0.835
**BMI**	32.7 (3.1)	108	34.0 (3.4)	400	<0.001
**QRISK2**	23.1 (10.1)	106	22.8 (9.3)	352	0.820
**% Male**	75 (69.4%)	108	262 (62.5%)	419	0.216

^a^Figures are mean (SD) for continuous data, or N(%) for categorical data. ^b^Not all surgeries were able to supply full demographic data for non-participants. ^c^Analyses are independent group t-tests for continuous data or Chi^2^ for categorical data.

### Exploratory analysis of outcomes

The following analyses (Tables 3 and 4) used last observation carried forward (LOCF) to impute missing data and the means are adjusted for baseline values of the outcome variable: The intervention group lost significantly more weight than controls at 4 months (Mean Diff −2.0Kg. 95%CI: −3.3 to −0.7, p = 0.001), but not at 12 months (Mean Diff −1.9Kg. 95%CI: −4.1 to 0.4, p = 0.103). No significant differences between groups were found in physical activity at either follow-up point. There were significant increases in self-reported fibre score and fruit and vegetable intake in the intervention group, compared with controls at both 4 and 12 months and changes in waist circumference (see Table 4) approached significance. Between group differences in other dietary intake variables were all in a direction commensurate with healthier eating.

**Table 3:** **Changes in weight and lifestyle behaviours**

**Variable**	**Group**	**Change 0–4 mth**	**N**	**Adjusted mean difference** ^**a**^	**Change 0–12 mth**	**N**	**Adjusted mean difference** ^**a**^
*Weight*							
Weight (Kg)	Intervention	−3.33 (3.48)	47	−2.46	−4.25 (5.49)	46	−2.38
				−3.86 to −1.06			−4.84 to 0.08
				p = 0.001**			p = 0.058
	Control	−0.99 (3.57)	49		−2.04 (6.87)	50	
Weight LOCF	Intervention	−2.85 (3.43)	55	−1.98	−3.65 (5.22)	55	−1.85
				−3.27 to −0.69			−4.08 to 0.38
							p = 0.103
				p = 0.003**			
	Control	−0.92 (3.44)	53		−1.90 (6.69)	53	
*Physical Activity (LOCF)*							
MVPA (min/day)	Intervention	1.79 (14.10)	53	−1.49	−0.51 (14.88)	53	−3.45
				−6.89 to 3.92			−9.38 to 2.48
							p = 0.251
				p = 0.587			
	Control	3.43 (14.60)	53		3.27 (18.82)	53	
Log MVPA (min/day)^b^	Intervention	0.019 (0.388)	53	−0.061	−0.021 (0.393)	53	−0.091
				−0.195 to 0.072			−0.262 to 0.080
	Control	0.065 (0.331)	53		0.049 (0.538)	53	
							p = 0.291
				p = 0.364			
Steps/day	Intervention	126.4 (1400)	53	−200	−141.0 (1903)	53	−345
							−1100 to 410
							p = 0.367
				−838 to 438			
				p = 0.535			
	Control	309.1 (1932)	53		166.1 (2291)	53	
Sedentary time (min/day)	Intervention	−11.9 (62.0)	53	13.3	−4.52 (56.05)	53	16.2
							−6.3 to 38.6
							p = 0.156
				−9.7 to 36.4			
				p = 0.255			
	Control	−16.0 (58.7)	53		−11.6 (61.4)	53	
Counts/minute of valid wear-time (Axis 1)	Intervention	15.0 (74.0)	53	−10.6	1.36 (74.52)	53	−25.3
							−57.0 to 6.3
							p = 0.116
				−38.8 to 17.6			
				p = 0.457			
	Control	22.5 (72.3)	53		19.0 (96.2)	53	
*Dietary intake (LOCF)*							
Fat score	Intervention	−5.76 (8.84)	54	−2.29	−5.00 (7.97)	54	−2.22
							−5.12 to 0.68
							p = 0.132
				−5.31 to 0.72			
				p = 0.134			
	Control	−4.50 (9.19)	52		−4.00 (10.34)	52	
Unsaturated fat score	Intervention	0.57 (1.80)	54	0.309	0.43 (1.81)	54	0.334
							−0.235 to 0.903
				−0.314 to 0.933			
							p = 0.247
	Control	0.63 (2.10)	51		0.51 (2.09)	51	
				p = 0.328			
Fibre score	Intervention	3.13 (8.86)	54	5.72	2.94 (10.87)	54	5.33
							1.83 to 8.82
							p = 0.003**
				2.80 to 8.65			
				p = 0.000**			
	Control	−2.69 (8.04)	51		−2.51 (10.02)	51	
Fruit & Vegetable score	Intervention	2.00 (5.62)	54	3.08	2.39 (7.08)	54	2.91
							0.65 to 5.16
							p = 0.012*
				1.10 to 5.07			
				p = 0.003**			
	Control	−0.98 (5.03)	51		−0.35 (5.48)	51	

^**a**^ANCOVA analysis with baseline value entered into the model. ^b^MVPA data were highly skewed, so analyses were repeated using log-transformed data. *p<0.05. **p < 0.01

**Table 4:** **Changes in biometric variables and quality of life at 12 months**

**Variable**	**Adjusted mean difference between groups (95%CI)** ^**a**^	
	**12 mths**	**N**
QRISK2 (10 year risk of CV event, %)	−0.76 (−2.19 to 0.66)	106
Systolic BP (mmHG)	1.09 (−3.67 to 5.85)	108
Diastolic BP (mmHG)	0.30 (−3.50 to 4.09)	108
Fasting glucose (mmol/l)	−0.13 (−0.29 to 0.04)	106
Fasting LDL (mmol/l)	0.15 (−0.08 to 0.37)	106
Fasting HDL (mmol/l)	−0.01 (−0.08 to 0.06)	107
Total cholesterol (mmol/l)	0.09 (−0.17 to 0.35)	107
Triglycerides (mmol/l)	−0.19 (−0.39 to 0.02)	107
Hba1c (mmol/l)	−0.84 (−1.89 to 0.21)	106
Waist (cm)	−2.18 (−4.43 to 0.06)^b^	108
BMI (Kg/m^2^)	−0.51 (−1.28 to 0.26)	108
ALT (IU/l)	0.92 (−2.43 to 4.27)	103
EQ5D	1.36 (−3.37 to 6.04)	107

^**a**^Based on ANCOVA analysis of LOCF data with baseline value entered into the model. ^b^p = 0.06

There were no significant changes in measures of blood pressure, cholesterol measures, blood glucose or other biometrics (see Table 4), or in quality of life (EQ-5D VAS overall health score). However, the prevalence of metabolic syndrome reduced in the intervention group, such that there was a significant difference in prevalence at 12 months (see Table 5), with a relative risk reduction of 28% in the intervention group and 2% in controls.

**Table 5:** **Changes in the prevalence of metabolic syndrome (using LOCF)**

	**N**	**% with metabolic syndrome**		**Pearson Chi** ^**2**^	**p value**
		**Intervention**	**Control**		
**Baseline**	106	53.7	61.5	0.67	0.438
**12 mth**	107	38.9	60.4	4.94	0.034

### Sensitivity analyses

**Figure 3:**
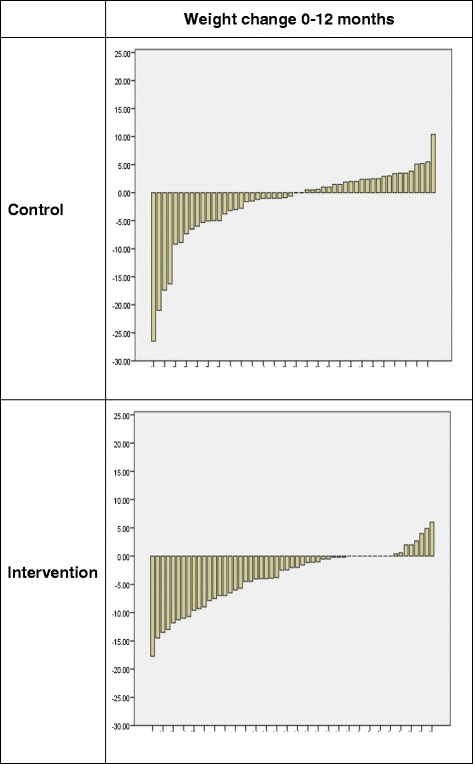
**Pattern of weight loss for individual participants.**

The pattern of change scores in each group is shown in Figure 3. This shows that very few people in the intervention group gained weight over the 12 month study period. However, in the control group, similar numbers of people gained and lost weight and the mean weight loss was dominated by the success of four individuals, who each lost over 15Kg.

The results were sensitive to co-interventions and co-morbidities, as follows: More controls (n=7) than intervention group participants (n=1) developed illnesses or took medications that could affect weight. More controls (n=6) than intervention group participants (n=2) engaged in other weight loss programmes during the course of the study. When these potential influences were entered into the analysis as covariates, there was a significant difference in weight loss between groups at 12 months, favouring the intervention group (Mean Diff 2.6Kg. 95%CI: −4.8 to −0.3, p = 0.025).

However, the results were not sensitive to differences between characteristics at baseline. Analyses including baseline values for age, diastolic blood pressure and sedentary time as covariates (either together or separately), or including the minimisation variables (BMI, pre-diabetes status and gender) found that none of these variables had a significant covariate effect (p > 0.1 in all cases).

A complete-case analysis of the data (including only people who provided data at both time points) showed that the intervention group lost significantly more weight then controls at 4 months (Mean Diff 2.5Kg: 95%CI: −3.9 to −1.1, p = 0.001, N = 96). The difference at 12 months approached, but did not reach significance (Mean Diff 2.4Kg: 95%CI: −4.8 to 0.1, p = 0.06, N = 96).

### Other relevant data

The correlation between baseline weight and weight at 12 months was 0.88 (p < 0.001). The intra-cluster correlation coefficient (ICC) for clustering of weight loss (0–12 months) by GP practice was 0.000 (95% CI 0.000, 0.084) for the whole sample (and was similar when examined within each group).

#### Attendance

Participants attended between zero and nine sessions (median = 7), with 70% of participants attending 5 or more of the 9 sessions. Attendance tapered off over time, particularly after session 6 (the attendance rates from sessions 1 to 9 respectively were 86%, 80%, 79%, 75%, 73%, 74%, 55%, 59%, 64%). Those attending 5 or more sessions lost 3.7Kg more at 4 mths than those attending less than 5 sessions (95%CI: 2.0 to 5.5, p < 0.001) and 4.1Kg more at 12 mths (95%CI: 1.2 to 7.1, p = 0.007).

#### Acceptability and feasibility

Feedback from questionnaires and participant interviews suggested a high level of satisfaction with the intervention. The fidelity checks showed good overall intervention fidelity, with some variation between providers. However, we identified a number of ways that we could improve intervention delivery and the content of the intervention and these will be reported in our quantitative and qualitative process evaluation reports.

### Cost

The intervention cost was estimated to be £310 per participant. This is based on delivery to groups of 10 people with 2 lifestyle coaches per group. A detailed breakdown of costs is provided in Additional file 3.

## Discussion

### Summary of findings

In this study we established the feasibility of conducting a full-scale trial of the Waste the Waist intervention. We successfully recruited a population with high cardiovascular risk, the intervention sessions were well attended and there is a strong signal that the intervention has the potential to be effective. People receiving the intervention lost an average 3.3Kg at 4 months and 4.3Kg at 12 months, demonstrating an encouraging weight maintenance profile. The control group lost more weight than expected, but this was largely explained by the performance of four individuals. Weight loss in the control group was mediated by the uptake of other weight loss programmes and co-morbidities. When these were taken into account, the difference between groups was significant at both time-points. Furthermore, increased exposure to the intervention was associated with a considerable increase in weight loss.

Changes in biometric risk markers were not significant, but the study was not powered to detect such changes. However, the presence of metabolic syndrome was reduced significantly at follow up in the intervention group compared with controls. This may reflect increased sensitivity to change in weight for this composite outcome and differences in the pattern of change in individuals (most intervention group participants lost some weight, but changes in the control group were concentrated in only a few individuals).

The level of weight loss achieved here (between 1.9 and 2.6 Kg more than controls at 12 months, depending which covariates are taken into account) compares well with established weight loss programmes used in primary care, including commercial weight loss programmes. These typically achieve 2 to 3 Kg of weight loss compared with usual care at 12 months of follow-up based on similar intention-to-treat analyses [19,31]. However, a full-scale trial in a larger sample is needed to produce an accurate estimate of effectiveness and cost-effectiveness.

Our intervention also seems to have a good weight maintenance profile compared with other weight loss interventions, which typically show a tendency towards weight regain following the initial intervention period [50]. This may reflect the fact that we offered (tapered) support for up to 9 months, or it may reflect our specific efforts to teach participants skills for sustainable management of their weight. The longer term maintenance profile (beyond 12 months) remains to be established.

The lack of any discernible impact on physical activity is a concern, given that this was one of the key behaviour change targets of the intervention. This may be due to a lack of attention to the process of translating motivation into practice by building a sense of competence to perform the target activities and to address concerns about safety of exercising, or about likely enjoyment. To do this we could incorporate more techniques such as setting up prompts/cues, prompting practice, or setting up rewards contingent on progress, which have recently been associated with increased physical activity in obese populations [51]. Modelling or demonstrating the intended exercise may also help to promote the take up of physical activity in older people [23,52]. These ideas are reinforced by data from our qualitative and quantitative process evaluation (to be reported elsewhere), suggesting that incorporation of practical demonstration/practice sessions, providing links to local activity-provision service and encouraging co-attendance of local activities by two or more group members might help to increase engagement with the physical activity component of the intervention.

### Strengths and limitations

The main strength of this study is the use of rigorous methods to assess the feasibility of conducting a full-scale randomised controlled trial and the conduct of exploratory analyses of the potential efficacy of the intervention. We used objective methods where possible to assess outcomes, including weight and physical activity. However, the study has some limitations: The randomisation resulted in a good balance between groups in terms of initial BMI, cardiovascular risk and gender. However, there were significant differences in age (as well as diastolic blood pressure and sedentary time, which may be related to age). We would therefore propose to add age as a minimisation variable in the subsequent full-scale trial. Methods for imputing missing data are widely debated [53] and we may consider using alternative imputation methods for the main trial. However, given the overall weight loss profile (increasing weight loss over time), LOCF seems a reasonable imputation method. Due to the small sample size, the large number of analyses conducted and the exploratory nature of this study, no definitive conclusions can be drawn about differences between groups for any of the outcomes measured. Although the care team was blinded to group allocation, Controls may have discussed this with their GPs and may have been more likely to seek out or take up offers of alternative interventions once they knew they were in the control group. Hence, given the increasing availability of community based weight loss interventions (both commercial and via referral from general practice), future studies should plan to monitor this and include it as a covariate in the primary analysis. Being from a single, relatively affluent locality, the sample was not representative of the wider UK population, particularly in terms of ethnicity and socio-economic status (i.e. deprivation index). Replication of the study in a larger and more diverse sample is therefore needed to establish the effectiveness of the intervention in a wider range of populations.

It is worth noting the high correlation between baseline and follow up weight. This may reflect the large proportion of relatively invariant mass in the body (from bone and organs, as opposed to fat) and is an important consideration for sample size calculation in weight loss studies. Because of the high degree of invariant mass, sample size should be based on the standard deviation for change in weight or on ANCOVA models with the correlation between baseline and follow-up taken into account.

### Implications for practice/future research

The proposed intervention has the potential to impact on the health of those at high risk of cardiovascular disease, and on NHS resource use. There is a high level of interest currently amongst primary care clinicians and commissioners about how to effectively support lifestyle change for cardiovascular risk management. If the generalisability and effectiveness are established in a full-scale trial, the intervention could also be adapted for people with diabetes and/or cardiovascular disease.

## Conclusions

The Waste the Waist intervention is deliverable in UK primary care, has acceptable recruitment and retention rates and, although this was only its first use, it seems to have a good potential for delivering clinically meaningful levels of weight loss. Subject to changes needed to the physical activity component and other changes suggested by our process evaluation (reported elsewhere), the intervention is now ready for evaluation in a full-scale trial.

## Additional files

Additional file 1:
**CONSORT statement for this article.**
Additional file 2:
**Intervention description.** The Waste the Waist Intervention. Additional file 3:
**Intervention costs for the Waste the Waist intervention.**
