# Supplementary material for: Waste the waist: a pilot randomised controlled trial of a primary care based intervention to support lifestyle change in people with high cardiovascular risk
Source: Int J Behav Nutr Phys Act. 2015 Jan 16;12:1. doi: 10.1186/s12966-014-0159-z (PMC4304605; doi:10.1186/s12966-014-0159-z)
Supplement: Additional file 2: — Intervention description. The Waste the Waist Intervention. [file 12966_2014_159_MOESM2_ESM.pdf]

The Waste the Waist Intervention

The Waste the Waist intervention was based on the Australian “Greater Green Triangle” (GGT) Programme.[1] This programme was selected as it a) produces more than 2Kg mean weight loss in people with elevated cardiovascular risk b) modifies other cardiovascular risk factors (plasma glucose, cholesterol, triglycerides, blood pressure) c) can be successfully delivered on a large scale[2] d) is within a cost threshold set by our primary care and public health stakeholders (less than £400 per person) e) includes, or can be modified to include, additional intervention components recommended by relevant systematic reviews[3-5] and advice from our service user advisory group and f) has a clearly defined theoretical basis.[6]

Aims: The intervention aimed to encourage weight loss by increasing physical activity, reducing intake of total and saturated fat, increasing fibre intake and other dietary changes (such as reducing portion sizes). Targets were set by participants, but the health benefits of 5% weight loss and of 150 mins per week of moderate activity were presented and suggested as minimum long-term targets for health gain.

Theoretical model: We extended the intervention and its theoretical model (the Health Action Process Approach[7]) by adding a number of behaviour change strategies to include a greater emphasis on social support, self-monitoring and relapse management and the use of coping plans.[8] Following a suggestion from our service-user group, we also added a novel element - explaining the process of behaviour change to participants. The process model for the Waste the Waist intervention is illustrated in Fig.1. The intervention processes involved a) increasing motivation (defined as perceived importance of healthy lifestyle, self-efficacy for

achieving healthy lifestyle, perceived risk and outcome expectations); b) making a specific action plan (including plans for social support and for overcoming barriers (coping plans)) and c) supporting maintenance through repeated ‘self-regulatory cycles’ of feedback /reflection, use of self-monitoring and relapse prevention techniques and revision of action plans.

Content: An overview of the content and timings for each session is provided in Appendix 1 below. The behaviour change techniques used to promote changes in the targeted processes are illustrated in Fig.2. These included motivational interviewing, self-assessment of risk factors (diet and physical activity levels), identifying and engaging sources of social support /addressing social influences, SMART-R goal setting (Specific, Measurable, Achievable, Relevant, Time-related, Repeatable), making coping plans and social support plans, self-monitoring of outcomes (using self-weighing and the participants’ choice of pedometers or physical activity diaries), frequent reviews of progress, problem-solving and goal review. We had a strong emphasis on empowering participants to develop and practice skills for lifestyle behaviour change. This included discussing how the process of behaviour change works and why they were being asked to engage in the different activities (such as action planning and self-monitoring). Thus, we made the model we were using (Fig.1) explicit from the outset.

To promote sustainability of weight loss we advised participants to make a series of small, achievable changes, rather than dramatic, unsustainable changes. We encouraged participants to prioritise ideas for change that would not detract from their enjoyment of food (for dietary changes) or that would be enjoyable or easy to

build into a routine (for physical activity).[9] Key messages that were repeated throughout the programme were “Make changes you can live with”, “Small changes make a big difference” and “Aim for a lifestyle that is both healthy and enjoyable”. We also included one technique based on cognitive behavioural therapy (using a mental ‘STOP’ sign and some pre-conceived strategies to address ‘in the moment’ food cravings).

Throughout the intervention we provided and discussed information about

a) Healthy eating - we used the Public Health England “Eat Well Plate”[10] as a guide and intervention tool to prompt ideas for reducing calorie intake by replacing fat (especially saturated fat) with fruit, vegetables and high fibre carbohydrates, reducing intake of high fat /high sugar snacks and reducing overall calorie intake.

b) Physical activity – including how to gauge intensity, especially in relation to walking pace and discussing ideas and opportunities for increasing physical activity. A set of home-based resistance exercises (as per government guidance to engage in muscle-strengthening exercise involving all muscle groups on at least two days of the week[11]) was introduced and discussed. However, no demonstration or structured exercise classes were provided.

Targets were set by participants, but the health benefits of 5% weight loss and of 150 minutes of moderate intensity physical activity were presented and suggested as minimum long-term targets for health gain.

Training and delivery: The style of delivery was considered to be important and we trained our lifestyle coaches to use person-centred counselling techniques derived from motivational interviewing (open questioning, affirmation, reflective listening, summaries, use of the elicit-provide-elicit (e-p-e) technique for information exchange)[12, 13] to deliver all of the intervention content. We recruited seven lifestyle coaches from the local community with varied backgrounds and experience, including group-based counselling (1), academic qualifications in nutrition or physical activity (2) and fitness industry /lifestyle coaching (4). A 2.5 day training course was developed and delivered by the co-authors (primarily CG, FG, AS).

We used session plans and slide-sets for each session to structure the intervention and used icons (pause buttons, flipchart symbols) to remind the lifestyle coaches to stop the slideshow and engage in discussion and other interactive activities specified in the session plan. Written manuals and materials were produced to support (and document) both the intervention and its training course.

The Waste the Waist intervention was delivered in local community venues (e.g. community halls, meeting rooms in GP practices after hours). The intervention consisted of four 120-minute group based sessions in the first month to support initial behaviour change, then five 90-minute maintenance support sessions at 1.5, 2, 4, 6 and 9 months after the first session. The total contact time was therefore 13.5 hours spread over 9 months. Groups consisted of 8-12 participants, facilitated by two lifestyle coaches.

Fig.S1: The Process Model of Lifestyle Behaviour Change.[14-16]

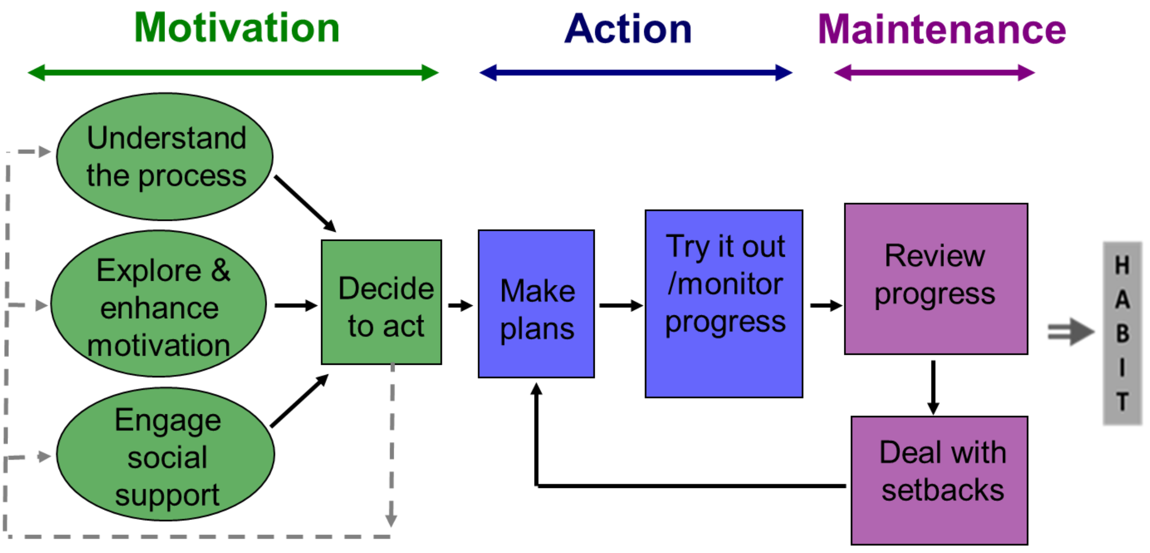

Over-arching philosophy:
Emphasise empowerment / ownership of goals and actions
Develop tools, strategies and motivation to manage lifestyle in the long term

Fig.S2: Behaviour change techniques used in Waste the Waist

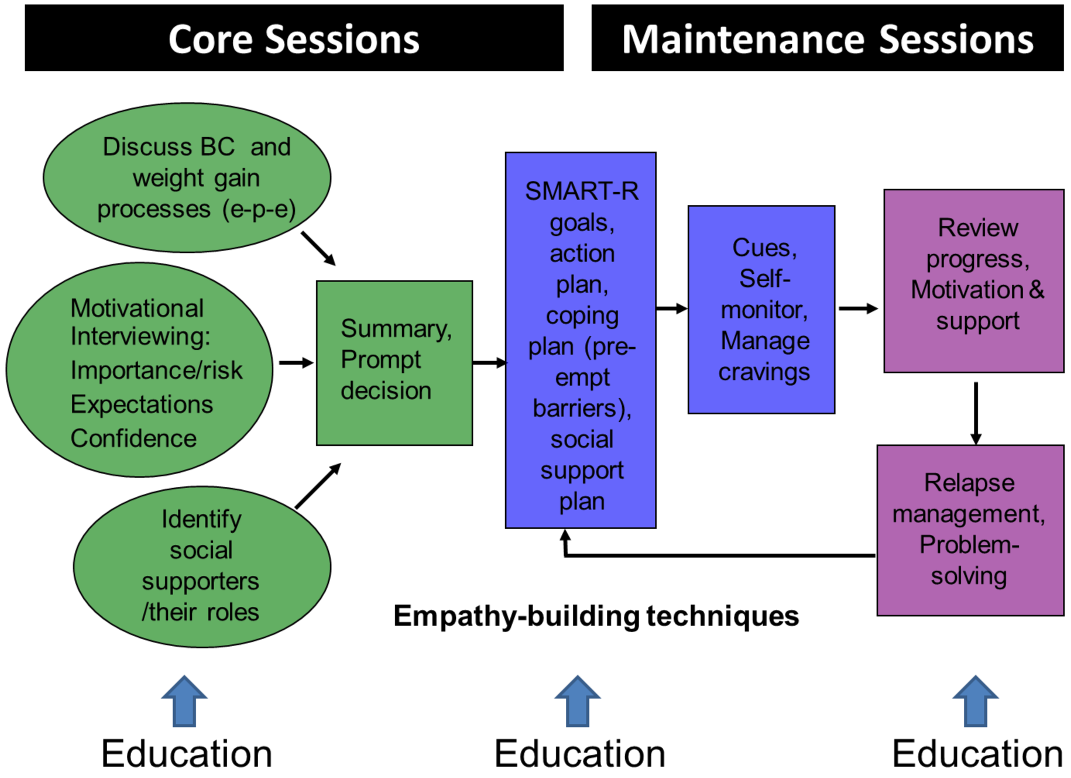

1. Laatikainen T, Dunbar J, Chapman A, Kilkkinen A, Vartiainen E, Heistaro S, Philpot B, Absetz P, Bunker S, O'Neil A, et al: **Prevention of Type 2 Diabetes by lifestyle intervention in an Australian primary health care setting: Greater Green Triangle (GGT) Diabetes Prevention Project.** *BMC Public Health* 2007, **7:**249.
2. Dunbar JA, Jayawardena A, Johnson G, Roger K, Timoshanko A, Versace VL, Shill J, Philpot B, Vartiainen E, Laatikainen T, et al: **Scaling up diabetes prevention in Victoria, Australia: policy development, implementation and evaluation.** *Diabetes Care* 2013.
3. Greaves CJ, Sheppard KE, Abraham C, Hardeman W, Schwarz P, Roden M, The IMAGE Study Group: **Systematic review of reviews of intervention components associated with increased effectiveness in dietary and physical activity interventions.** *BMC Public Health* 2011, **11:**1-12.
4. Dombrowski SU, Sniehotta FF, Avenell A, Johnston M, MacLennan G, Araújo-Soares V: **Identifying active ingredients in complex behavioural interventions for obese adults with obesity-related co-morbidities or additional risk factors for co-morbidities: a systematic review.** *Health Psychology Review* 2012, **6:**7-32.
5. Artinian NT, Fletcher GF, Mozaffarian D, Kris-Etherton P, Van Horn L, Lichtenstein AH, Kumanyika S, Kraus WE, Fleg JL, Redeker NS, et al: **Interventions to promote physical activity and dietary lifestyle changes for cardiovascular risk factor reduction in adults: A scientific statement from the American Heart Association.** *Circulation* 2010, **122:**406-441.
6. Uutela A, Absetz P, Nissinen A, Valve R, Talja M, Fogelholm M: **Health psychological theory in promoting population health in Paijat-Hame, Finland: First steps towards a type 2 diabetes prevention study.** *J Health Psychol* 2004, **9:**73-84.
7. Schwarzer R: **Self-efficacy in the adoption and maintenance of health behaviors: Theoretical approaches and a new model.** In *Self-efficacy: Thought control of action.* Edited by Schwarzer R. Washington DC: Hemisphere Publishing Corp; 1992.
8. Gillison FB, Greaves CJ, Stathi A, Ramsay R, Bennett P, Taylor G, Francis M, Chandler R: **"Waste the Waist": The development of an intervention to promote changes in diet and physical activity for people with high cardiovascular risk.** *Br J Health Psychol* 2012, **17:**327-345.
9. Davis M, Fox K, Hillsdon M, Coulson J, Sharp D, Stathi A, Thompson J: **Getting out and about in older adults: the nature of daily trips and their association with objectively assessed physical activity.** *Int J Behav Nutr Phy* 2011, **8:**116.
10. NHS Choices: **The eatwell plate.** http://www.nhs.uk/Livewell/Goodfood/Pages/eatwell-plate.aspx: UK Department of Health; 2013.
11. Health Do: **Start Active, Stay Active: A report on physical activity for health from the four home countries' Chief Medical Officers.** London; 2011.
12. Rollnick S, Mason P, Butler C: *Health Behaviour Change: A guide for practitioners.* Edinburgh: Churchill Livingstone; 1999.
13. Miller WR, Rollnick S: *Motivational interviewing: Preparing people for change (2nd ed).* New York: Guildford Press; 2002.
14. Lindström J, Neumann A, Sheppard K, Gilis-Januszewska A, Greaves CJ, Handke U, Pajunen P, Puhl S, Pölönen A, Rissanen A, et al: **Take Action To Prevent Diabetes: A toolkit for the prevention of type 2 diabetes in Europe.** *Horm Metab Res* 2010, **42:**S37-S55.
15. Greaves CJ, Reddy P, Sheppard K: **Supporting behaviour change for diabetes prevention.** In *Diabetes Prevention in Practice.* Edited by Schwarz P, Reddy P, Greaves CJ, Dunbar J, Schwarz J. Dresden: TUMAINI Institute for Prevention Management; 2010: 19-29
16. Greaves CJ: **Supporting behaviour change in general practice.** In *Practical Psychology in Diabetes Care.* Edited by Barnard K, Lloyd CE. London: Springer-Verlag; 2012.

APPENDIX 1: Session Content

Session 1: Developing motivation

Objectives:

- **To introduce group members to one another, and promote *active patient involvement* and positive group interactions**
- **To promote Waste the waist participants' understanding that they are at high risk of developing heart disease and/or diabetes and would benefit from making lifestyle changes**
- **To prepare participants for lifestyle change by helping them to understand *how* to make lasting behaviour change**

Main Messages

– Changing your diet and physical activity will reduce your risk of cardio-vascular disease and type 2 diabetes

– Small changes can make a big difference to your weight and your health

– If you follow this programme, you will learn how to manage your lifestyle to make and sustain changes for life.

– Aim for a lifestyle that is both healthy and enjoyable - make changes that you can live with!

Pre session reading: Facilitators are advised to be particularly familiar with the session plan and Powerpoint slideshow for Session 1 and the following Session Materials:

Session materials needed:

- Sheet 1.0: Register
- Sheet 1.1: How the group should work
- Sheet 1.2: Process of behaviour change.
- Sheet 1.3: Myth busting
- Sheet 1.4: Programme goals
- Sheet 1.5: Decisional balance sheet
- Sheet 1.6: Importance ruler
- Sheet 1.7: Social support sheet.
- Sheet 1.8: Physical activity diary
- Sheet 1.9: End of session checklist

Additional materials:

- Flipchart stand, paper pads, coloured flipchart pens (2-3 sets needed)
- Laptop and powerpoint projector, extension lead
- Pedometers and pens for participants
- Digital recorder
- 10-12 Participant Manuals – one each

- Expense claim sheets for participant travel expenses

	Timings
**Outline of topics and timings**	
1. Welcome and introduction	10 min
2. Working together: Group guidelines	8 min
3. Understanding behaviour change	13 min
4. Overview of CVD risk	20 min
SHORT BREAK	10 min
5. Programme goals	2 min
6. Getting motivated	20 min
7. Preparing for lifestyle change: social support	15 min
8. Take Away Tasks	5 min
9. Reflection on today's session	1 min
	104 min

Session 2: Getting Going

Objectives:

- **To encourage positive group interactions and active participant engagement with the facilitator, with each other and with the programme.**
- **To exchange information about what constitutes an active lifestyle and how physical activity is linked to CV risk.**
- **To help participants assess their physical activity levels and compare them with the current physical activity recommendations.**
- **To make a first steps action plan for increasing physical activity including goals, a coping plan and a plan for social support.**
- **To further increase participants' understanding of the process of behaviour change and the strategies Waste the Waist uses to help them succeed in making changes in diet and physical activity.**
- **To increase perceived importance of making changes to physical activity**
- **To increase perceived confidence about increasing physical activity**

Main Messages

- Being physically active can make a real difference to your risk of heart disease
- Moderate activity is ideal, but any increase in physical activity is beneficial.
- There are many different ways that you can incorporate more physical activity in your daily routine.
- Aim for a lifestyle that is both healthy and enjoyable - Make changes that you can live with.

Pre session reading:

Facilitators are advised to be familiar with this session plan, the Powerpoint slideshow for Session 2 and the following Session Materials:

Session materials needed:

- Sheet 2.0: Sign-up sheet
- Sheet 1.2: Process of behaviour change
- Sheet 1.4: Programme goals
- Sheet 1.6: Importance ruler
- Sheet 1.7: Social support sheet
- Sheet 1.8: Physical activity diary
- Sheet 2.2: Physical activity guidelines
- Sheet 2.3: Decisional Balance worksheet
- Sheet 2.4: Confidence ruler
- Sheet 2.5: Action planning sheet (PA action plan and Example PA action plan)
- Sheet 2.6: Barriers informational sheet
- Sheet 2.7: Food diary (for take away task)
- Sheet 2.8: Pros and cons of getting more active checklist (for Lifestyle Coach)
- Sheet 2.9: SMART goal setting
- Sheet 2.10: End of session checklist

Additional materials:

- Weighing scales
- Flipchart – stand, paper pads, coloured flipchart pens (2-3 sets needed)
- Laptop and powerpoint projector
- Pedometers for participants
- Digital recorder
- 1-2 Spare participant manuals
- Expense claim sheets for participant travel expenses

Outline of topics and timings	Time
1. Welcome, review session 1, feedback on last week's social support task, state objectives of today's session	10 mins
2. Overview of physical activity	20 mins
3. How much are you getting?	10 mins
4. Assess importance of physical activity	10 mins
5. Assess confidence	10 mins
SHORT BREAK	10 mins
6. Action planning: Setting SMART goals	15 mins
7. Action Planning: Social support	5 mins
8. Action Planning: Relapse prevention	10 mins
9. Keeping Track	3 mins
10. Take Away Tasks	2 mins
11. Reflection on session 2	2 mins
12. Overview of next session	1 mins
13. Weight measurement (optional) /Q&As	10 mins
	108 mins (+10 weight measurement)

Session 3: Healthy Eating

Objectives:

- **To encourage positive group interactions and active participant engagement with the facilitator, with each other and with the programme.**
- **To exchange information about what constitutes a healthy diet (as defined by the Eat Well Plate) and how healthy eating is linked to CV risk.**
- **To help participants assess their dietary habits and compare them with the Eat Well Plate recommendations.**
- **To make a "first steps" action plan for healthier eating including goals, a coping plan and a plan for social support.**
- **To further increase participants' understanding of the process of behaviour change and the strategies Waste the Waste uses to help them succeed in making changes in diet and physical activity.**
- **To increase perceived importance of making changes to diet.**
- **To increase perceived confidence about making changes to diet.**

Main Messages

– Healthy eating can make a real difference to your weight and to your risk of heart disease.

– A healthy diet is one where you limit the amount of fat and saturated fat, limit the amount of sugary drinks and snacks, get plenty of fruit and veg and plenty of starchy foods (especially those that are wholegrain).

– Changing the balance of things on your plate, changing the type of foods you choose (e.g. lower fat or wholegrain options) and changing the way you cook food are three ways to make your diet healthier.

– Aim for a diet that is both healthy and enjoyable!

Pre session reading:

Facilitators are advised to be particularly familiar with this session plan, the Powerpoint slideshow for Session 3 and the following Session Materials:

Session materials needed (Participant Manual):
- Sheet 3.0: Register
- Sheet 1.6: Importance ruler – Healthy eating
- Sheet 1.7b: Social support checklist
- Sheet 2.4: Confidence ruler – Healthy eating
- Sheet 3.0: Session 3 sign up sheet
- Sheet 3.2: Healthy Eating Checklist
- Sheet 3.4: Diet MOT Part 1 Slides as a handout
- Sheet 3.5: Fat and Fibre Quiz for Take Away task:
- Sheet 3.9: Healthy Eating Action Plan

- Sheet 3.10 End of session checklist

Additional materials (Facilitators):

- Weighing scales
- Flipchart – stand, paper pads, coloured flipchart pens (2-3 sets needed)
- Laptop and powerpoint projector
- 6 rulers (or other dividers for the Eatwell task)
- 2-3 Spare Food diaries
- 1-2 spare pedometers
- Digital recorder
- 1-2 Spare participant manuals
- Expense claim sheets for participant travel expense

Outline of topics and timings	Time
14. Introduction: Welcome back and recap session 2; state objectives of today's session; review progress with take away tasks (physical activity plans (briefly); walking pace)	8 mins
15. Overview of healthy eating	15 mins
16. Assess Importance of healthy eating	5 mins
17. Eating Well: What is a healthy balanced diet?	15 mins
SHORT BREAK	10 mins
18. Diet MOT	20 mins
19. Action planning: Setting SMART goals and Keeping track	10 mins
20. Action Planning: Social support	5 mins
21. Action Planning: Assess Confidence and Coping plans	10 mins
22. Take Away tasks	1 mins
23. Reflection on session 3	1
24. Overview of next week	1
25. Weight measurement (optional)	10 mins
	101 mins (+10 weight measure)

Session 4: Dealing with temptations and challenges

Objectives:

- **To encourage positive group interactions and active participant engagement with the facilitator, with each other and with the programme.**
- **To reinforce messages about what healthy eating is**
- **To practise food label reading and discuss ways to choose healthier alternatives when shopping.**
- **To discuss the temptations and challenges that can put our healthy eating plans at risk when we eat out and to identify coping strategies.**
- **To discuss the link between food and mood**
- **To review progress with and update the "first steps" action plan for healthier eating.**
- **To review progress with and update the "first steps" action plan for physical activity.**
- **To further increase participants' understanding of the process of behaviour change and the strategies Waste the Waste uses to help them succeed in making changes in diet and physical activity.**

Main Messages

- Changing the balance of things on your plate, changing the type of foods you choose (e.g. lower fat or wholegrain options) and changing the way you cook food are three ways to make your diet healthier and to reduce the overall amount of calories you eat.
- Small changes can make a big difference
- Ways to control your appetite might include recognising and challenging automatic eating when it occurs – this involves putting up a mental STOP sign and trying to think of the healthy option in each situation.
- Finding alternative ways to manage stress or moods might also help you get better control over your appetite.
- Make changes that you can live with – aim for a lifestyle that is healthy and enjoyable.

Pre session reading:

Facilitators are advised to be particularly familiar with this session plan, the Powerpoint slideshow for Session 3 and the following Session Materials:

Session materials needed:

- Sheet 1.4: Programme goals
- Sheet 1.6: Importance ruler
- Sheet 2.3: Decisional balance sheet
- Sheet 2.4: Confidence ruler
- Sheet 2.7: Spare Food diaries

- Sheet 3.5: Fat and Fibre Quiz
- Sheets 3.9: Healthy Eating Action Plan
- Sheet 4.0: Sign up sheet
- Sheet 4.1 and 4.1a: Healthy swaps worksheet
- Sheet 4.2: Food Label Reading
- Sheet 4.3: Managing Food Cravings
- Sheet 4.4: Mindfulness
- Sheet 4.5: Small Changes
- Sheet 4.6: End of session checklist

Additional materials:

- **Shopping bag with 15-20 items (or food labels)**
- Weighing scales
- Flipchart – stand, paper pads, coloured flipchart pens (2-3 sets needed)
- Laptop and powerpoint projector
- 6 rulers (or other dividers for the Eatwell task)
- 1-2 spare pedometers
- 2-3 spare healthy eating action plans
- Digital recorder
- 1-2 Spare participant manuals
- Expense claim sheets for participant travel expenses

Outline of topics and timings	Time
26. Welcome, recap session 3, state objectives of today's session	2 min
27. Review and update of physical activity action plans	15 min
28. The Fat and Fibre quiz – another tool for checking your diet	10 min
29. Label reading and shopping bag: What do we put in our basket?	10 min
30. Small changes make a big difference (and recipe modification)	10 min
SHORT BREAK	10 min
31. Eating out: Social influences	10min
32. Managing Food Cravings	10min
33. Update healthy eating action plans	20 min
34. Homework	2 min

35. Reflection on session 4	1 min
36. Overview of next week	2 min
37. Weight measurement (optional) with Q&A time in parallel	10 min
	90 min (+10 for weighing)

Session 5: Maintaining Motivation

Objectives: **In this session, the participant will**

- **Review their progress with their existing 'first steps' action plans**
- **Identify any barriers to change and ways to overcome these barriers**
- **Review their motivations for making lifestyle changes**
- **Review their goals and consider adding to them (working towards longer-term goals)**
- **Review their efforts at engaging social support**
- **Revise their action plans accordingly**

Main Messages

- Aim for a lifestyle that is both healthy and enjoyable
- Make changes that you can live with
- Keep track of your progress;
  - using self-monitoring tools (like diaries or your pedometer) will help you to make sure you're maintaining changes in your lifestyle, and not slipping back to old ways
  - comparing progress against your long-term targets will help you see whether the goals you've chosen are working for you
- Tackle set-backs head on - if things aren't going well, take stock and try out different coping strategies until you find what works for you
- Challenge negative thinking patterns

Pre session reading:

Facilitators are advised to be particularly familiar with this session plan and the Powerpoint slideshow. It may also be useful to refresh your memory of action planning from its first introduction in Session 2.

Session materials needed:

- Sheet 5.0: Register
- Sheet 5.1: Progress review chart
- Sheet 5.2: Checklist for promoting enjoyment
- Sheet 5.4: End of session checklist

Additional materials:

- Weighing scales
- Fresh Action Plans for both diet and physical activity
- Laptop and projector for Powerpoint presentation
- 2-3 Pedometers for participants (to replace any lost)
- Flipchart – stand, paper pads, coloured flipchart pens (2-3 sets needed)
- Digital recorder
- 1-2 Spare participant manuals
- Expense claim sheets for participant travel expenses

Outline of topics and timings		Time
(Pre-session: 10 min catch-up for those who missed last session)		
38.	Welcome, briefly review key messages from session 4	5 mins
39.	Review progress with diet and PA, celebrate success, re-frame failure	15 mins
40.	Problem solving & coping strategies	20 mins
41.	Positive thinking	15 mins
SHORT BREAK		10 min
42.	Revising action plans	18 mins
43.	Re-visiting self-monitoring	4 mins
44.	Take away tasks	2 mins
45.	Overview of next session	2 mins
46.	Weight measurement (optional) with Q&A time in parallel	10 mins
	Total:	*91 (+10 min optional)*

Session 6: Keeping Going (I)

Objectives: **In this session, the participant will**

- **Review their progress with their existing diet and physical activity plans, celebrate success and reframe failure**
- **Review their motivations for making lifestyle changes (and amend action plans)**
- **Have an opportunity to discuss healthy eating with a dietitian**
- **Reinforce the importance of self-monitoring**
- **Review their goals and consider adding to them (working towards longer-term goals)**
- **Revise their action plans accordingly**

Main Messages

- Make changes that you can live with
- Keep track of your progress
  - Using self-monitoring tools (like diaries or the pedometer) will help you to make sure you're maintaining changes in your lifestyle, and not slipping back to old ways
  - Comparing progress against your long-term targets will help you see whether the goals you've chosen are working for you
- Tackle set-backs head on - if things aren't going well, take stock and try out different coping strategies until you find what works for you.

Pre session reading:

Facilitators are advised to be particularly familiar with this session plan, the Powerpoint slideshow for Session 6 and the following Session Materials:

Session materials needed:

- Sheet 6.0: Register
- Sheet 6.4: End of session checklist

Additional materials:

- Weighing scales
- Fresh Action Plans for both diet and physical activity
- Laptop and projector for Powerpoint presentation
- 2-3 Pedometers for participants (to replace any lost)
- Flipchart – stand, paper pads, coloured flipchart pens (2-3 sets needed)
- Digital recorder
- 1-2 Spare participant manuals
- Expense claim sheets for participant travel expenses

Outline of topics and timings		Time
(Pre-session: 10 min catch-up for those who missed last session)		
47.	Welcome, briefly review key messages from session 5	4 mins
48.	Review progress with diet and PA, celebrate success, re-frame failure	15 mins
49.	Meet the Expert! Discussion about healthy eating	40 mins
SHORT BREAK		10 min
50.	Problem solving & coping strategies	20 mins
51.	Revising action plans	15 mins
52.	Revisiting self-monitoring	4 mins
53.	Take away tasks	1 min
54.	Overview of next session	1 min
55.	Weight measurement (optional) with Q&A time in parallel	10 mins
	Total:	*110 (+10 min optional)*

Session 7: Getting Stronger

Objectives: In this session, the participant will

- **Review their progress with their existing diet and physical activity plans, celebrate success and reframe failure**
  - **Review their motivations for making lifestyle changes**
  - **Learn about and have an opportunity to discuss ideas about muscle strength training**
  - **Reinforce the importance of self-monitoring**
  - **Review their goals and consider adding to them (working towards longer-term goals)**
  - **Revise their action plans accordingly**

Main Messages

- Adding muscle strength exercises to your physical activity plan could have some strong benefits, including reducing your risk of heart disease, strokes and type 2 diabetes.
- Aim for a lifestyle that is both healthy and enjoyable
- Make ‘sustainable’ changes: ones that you can live with
- Keep track of your progress
- Tackle set-backs head on - if things aren’t going well, take stock and try out different coping strategies until you find what works for you.

Pre session reading: Facilitators are advised to be particularly familiar with this session plan, the Powerpoint slideshow for Session 7 and the following Session Materials:

Session materials needed:

- Sheet 7.0: Register
- Sheet 5.1: Progress review chart (in User Manual)
- Sheet 7.1: Working your muscles (in User Manual)
- Sheet 7.4: End of session checklist

Additional materials:

- Weighing scales
- Fresh Action Plans for both diet and physical activity
- Laptop and projector for Powerpoint presentation
- 2-3 Pedometers for participants (to replace any lost)
- Flipchart – stand, paper pads, coloured flipchart pens (2-3 sets needed)
- Digital recorder
- 1-2 Spare participant manuals
- Expense claim sheets for participant travel expenses

**Outline of topics and timings**		**Time**
(Pre-session: 10 min catch-up for those who missed last session)		
56.	Welcome, briefly review key messages from session 6	4 mins
57.	Review progress with diet and PA, celebrate success, re-frame failure	15 mins
58.	Getting Stronger: Muscle exercises and heart disease risk	30 mins
SHORT BREAK		10 min
59.	Are we having fun yet?	10 mins
60.	Problem solving & coping strategies	20 mins
61.	Revising action plans	15 mins
62.	Revisiting self-monitoring	4 mins
63.	Take away tasks	1 min
64.	Overview of next session	1 min
65.	Weight measurement (optional) with Q&A time in parallel	10 mins
	Total:	*110 (+10 min optional)*

Session 8: Keeping Going

Objectives: **In this session, the participant will**

- **Review their progress with their existing diet and physical activity plans, celebrate success and reframe failure**
  - **Discuss ways for managing stress**
  - **Review their motivations for making lifestyle changes**
  - **Review their goals and consider adding to them (working towards longer-term goals)**
  - **Revise their action plans accordingly**

Main Messages

- Make ‘sustainable’ changes: ones that you can live with
- Keep track of your progress
- Tackle set-backs - if things aren’t going well, take stock and try out different coping strategies until you find what works for you.
- You can use simple techniques to manage stressful events and situations.

Pre session reading:

Facilitators are advised to be particularly familiar with this session plan, the Powerpoint slideshow for Session 8 and the following Session Materials:

Session materials needed:

- Sheet 8.0: Register
- Sheet 5.1: Progress review chart
- Sheet 8.2: Stress management
- Sheet 8.3: Progressive relaxation worksheet
- Sheet 8.4: End of session checklist,

Additional materials:

- Weighing scales
- Fresh Action Plans for both diet and physical activity
- Laptop and projector for Powerpoint presentation
- 2-3 Pedometers for participants (to replace any lost)
- Flipchart – stand, paper pads, coloured flipchart pens
- Digital recorder
- 1-2 Spare participant manuals
- Expense claim sheets for participant travel expenses

Outline of topics and timings		Time
(Pre-session: 10 min catch-up for those who missed last session)		
66.	Welcome, briefly review key messages from session 7	4 mins
67.	Review progress with diet and PA, celebrate success, re-frame failure	15 mins
68.	Stress Management	30 mins
SHORT BREAK		10 min
69.	Problem solving & coping strategies	20 mins
70.	Revising action plans	20 mins
71.	Take away tasks	1 min
72.	Relaxation technique	10 mins
73.	Overview of next session	1 min
74.	Weight measurement (optional) with Q&A time in parallel	10 mins
	Total:	111 (+10 min optional)

Session 9: Into the Future

Objectives: **In this session, the participant will**

- **Review their progress in using the general process for making lifestyle changes**
- **Review their progress with their existing diet and physical activity plans (celebrate any successes and reframe failures)**
  - **Review their motivations for making lifestyle changes**
  - **Think about the longer term and their "possible future selves"**
  - **Review their goals and consider adding to them (working towards longer-term goals)**
  - **Discuss ways of maintaining weight loss and taking it further**

Main Messages

- Aim for a lifestyle that is both healthy and enjoyable
- Make changes that you can live with
- Keep track of your progress
- Try out different coping strategies until you find what works for you
- If you want to go further, add some more changes

Pre session reading:

Facilitators are advised to be particularly familiar with this session plan, the Powerpoint slideshow for Session 9 and the following Session Materials:

Session materials needed:

- Sheet 9.0: Register
- Sheet 5.1: Progress review chart
- Sheet 9.2a, 9.2b: Eating Out
- Sheet 9.4: End of session checklist

Additional materials:

- Weighing scales
- Fresh Action Plans for diet and physical activity
- Laptop and projector for Powerpoint presentation
- 2-3 Pedometers for participants (to replace any lost)
- Flipchart – stand, paper pads, coloured flipchart pens
- Digital recorder
- 1-2 Spare participant manuals
- Expense claim sheets for participant travel expenses

Contents		Time
(Pre-session: 10 min catch-up for those who missed last session)		
75.	Welcome, briefly review key messages from session 8	2 mins
76.	Review progress with diet and PA, celebrate success, re-frame failure.	10 mins
77.	Problem solving & coping strategies	10 mins
78.	Review progress with using the process of lifestyle change	10 mins
79.	Tailing off /keeping the ball rolling	15 mins
SHORT BREAK		10 min
80.	Choice of activities: Eating out or client-led activity /open discussion	15 mins
81.	Maintaining lifestyle changes	15 mins
82.	Revising action plans	15 mins
83.	Take away tasks and final farewell	2 mins
84.	Weight measurement (optional) with Q&A time in parallel	10 mins
	Total:	*104 (+10 min optional)*
