# Supplementary material for: Waste the waist: a pilot randomised controlled trial of a primary care based intervention to support lifestyle change in people with high cardiovascular risk
Source: Int J Behav Nutr Phys Act. 2015 Jan 16;12:1. doi: 10.1186/s12966-014-0159-z (PMC4304605; doi:10.1186/s12966-014-0159-z)
Supplement: Additional file 3: — Intervention costs for the Waste the Waist intervention. [file 12966_2014_159_MOESM3_ESM.pdf]

Table A3.1: Estimated resource use and costs for delivery of the Waste the Waist intervention per group of 10 participants.

Item of resource use	Mean resource use per group	Unit cost (£)	Mean cost (£, 2011)
Lifestyle Coaches (delivery of intervention, preparation of material, travel, supervision). Based on two lifestyle coaches per group.	92.66	£21/hour[1]	£1,945.86
Supervisory input	1.33 hours	£60/hour[2]	£80
Intervention venue	27 hours (9 sessions)	£20/hour	£540
Consumables	1 pedometer per participant	£10/pedometer	£100
Administrative support (telephone, emails and letters)	16 hours	£21/hour[3]	£336
Estimated total cost for delivery of intervention per group			£3001.86
Estimated total mean delivery cost per participant			£300.19
Set-up training costs-estimated mean cost per participant			£10.22
**Estimated total mean cost per participant**			**£310.41**

[1]Curtis (2012), Based on salary structure of a worker employed on NHS scale AfC Band 2 point 5, £21 (see Table 10.5, p179) Clinical support worker nurse (community) was used as a proxy as we assumed the salary structure to be similar to that of a HT. This estimate of cost (per hour) for HT includes salary on-costs, and management/capital overheads.

[2]Curtis (2012), Based on salary structure of a worker employed on NHS scale AfC Band 8a, per hour, £60 (see Table 9.5, p171). Clinical psychologist was used as a proxy as we assumed the salary structure to be similar to that of the Lead trainer (CG). This estimate of cost (per hour) for HT includes salary on-costs, and management/capital overheads.

[3]Curtis (2012), Based on the median salary structure of a worker employed on the NHS scale AfC Band, £21 per hour (see Table 10.5, p179) Clinical support worker (community) was used as a proxy as we assumed the salary structure to be similar to that of an administrative clerk.

Curtis, L. Unit costs of health and social care. Canterbury: Personal Social Services Research Unit, University of Kent; 2012.
